# "Eco-conscious appetites: Investigating organic food purchase intentions through consumption values, empowered by environmental self-identity and analyzed using MGA – Baltic insights"

**DOI:** 10.1016/j.heliyon.2024.e35330

**Published:** 2024-07-27

**Authors:** Aušra Rūtelionė, Muhammad Yaseen Bhutto

**Affiliations:** School of Economics and Business, Kaunas University of Technology, 44239, Kaunas, Lithuania

**Keywords:** Consumption values, Environmental self-identity, Multi-group analysis, Organic food, Baltic economy, Lithuania

## Abstract

Growing consumer interest in organic food has attracted the attention of the academic community. While the existing literature broadly examines the acceptability of organic food, there is a recognized need to delve deeper into consumer values. This study fills this gap by applying the theory of consumer values. The data was collected as part of a survey of 1000 Lithuanian consumers to evaluate the proposed hypotheses empirically. The results show that functional value (quality), social value, conditional value, emotional value, and epistemic value positively influence purchase intention for organic food. Conversely, functional value (price) does not significantly impact the purchase intention of organic food. Furthermore, the study recognizes a significant moderating impact of environmental self-identity in shaping the relationship between conditional value, emotional value, and purchase intention of organic food. In addition, a multi-group analysis examines notable differences between consumer groups based on gender, education, age, and income. The results indicate significant group differences, showing that purchasing behavior among women, younger consumers, those with higher incomes, and those with higher education levels are more susceptible to these values. This research improves our understanding of consumer values regarding organic food in Lithuania and provides valuable guidance to managers and policymakers. By recognizing the intricate interplay between different values and the influence of environmental self-identity, stakeholders can better tailor their marketing efforts and policies to meet the unique preferences of different consumer groups, ultimately promoting the growth of the organic food market.

## 1 Introduction

In light of rising global environmental issues like global warming, pollution, and ecological decline, sustainable consumption patterns have gained the utmost significance in consumer markets and research over recent decades [1]. These environmental harms can be solved by changing people's behavior to be more environmentally sustainable [2]. Consequently, organic food consumption has increased noticeably and significantly [3]. Organic food includes all foods grown, stored, and processed in soil enriched with natural fertilizers and without growth hormones, using artificial synthetic chemicals and GMOs [4,5]. The organic food market was US$188.35 billion in 2021 and is projected to grow, reaching US$208.19 billion by 2022 [6]. In the organic food product market, the European Union member states garnered the runner-up position globally, trailing solely behind the United States of America [7]. The organic foods market has experienced substantial growth; however, there is a significant research gap concerning this market's relative size and differentiation among diverse European countries. The varying levels of development observed in organic food markets across Europe emphasize the necessity for further exploration into the adoption of organic food within the European region [4]. Previous literature focuses predominantly on research conducted on organic foods in developed economies, including the United States and a significant portion of Western European countries such as Sweden, Greece, Germany, and Austria [4,[8], [9], [10]]. However, the research found organic food industry expansion in Baltic economies like Lithuania is comparatively slow, indicating that this sector is still at an early stage of progress [11]. The highest organic retail sales per capita in Europe is in Denmark, 227 euros per capita, while in Lithuania, this measure is only 2 euros per capita, and the average equivalent in Europe spent per capita is 60,5 euros, shows Lithuania is far behind the EU average [12]. Like other European countries, international and domestic brands aim to meet the demands of organic consumers in the Lithuanian organic food market. However, there is a lack of research into consumer behavior, especially the positive propensity to buy organic food, in emerging Baltic markets such as Lithuania.

Factors influencing the consumption of organic foods have been extensively studied. Among the factors that can influence people's organic buying behavior, studies have also reported the following factors as vital: consumers' purchase intention toward organic food [13,14], consumer attitude [15], health orientation [16], cultural dimensions [17], environmental orientation [18,19], subjective norms [20], organic cognition [21], motivational factors [22], available media information [23], food safety [24]. From the theoretical point of view, TPB is one of the most widely used social psychological models for predicting human behavior and intention in diverse contexts, especially in the context of consumer behavior toward organic food [13,25], primarily because it allows the model to be extended by incorporating other relevant independent variables to explain consumer intentions toward organic food [26,27]. Some researchers have attempted to examine organic food purchasing decisions through consumption values. Qasim et al. [28] conducted a study to examine how consumption values influence consumers' behavioral intentions. The results of this research showed a recognizable and direct association between conditional, emotional, and epistemic values and the propensity to choose organic foods for consumption. Waseti and İrfanoğlu [29] examined the influence of consumption values on intention concerning organic food. The study demonstrated the mediating influence of consumer participation in establishing the result between functional, social, and emotional values and purchase intention of organic food. Similarly, vein Mohd Suki et al. [30] found consumers' environmental concerns are significantly influenced by their social values. Additionally, the researchers found that the conditional value consumers hold significantly affects their decision-making process when purchasing organic foods. However, it is essential to recognize that various situational factors influence buying decisions, including product features, time constraints, availability, environmental factors, habits, government incentives, and marketing rebates and promotions [31]. Therefore, the preceding summary of relevant scholarly work clearly shows that scholars predominantly used the Theory of Planned Behavior (TPB), often with some adjustments or extensions, while giving only limited consideration to consumers' inherent values to understand the intention underlying the acquisition of organic food.

Some scholars pointed out that consumer self-identity positively correlated with sustainable purchase behavior [32,33]. Recent studies recommend that consumers exhibit environmental self-identity that may serve as valuable indicators for predicting an extensive range of pro-environmental actions [34]. Prior research mainly analyzed the direct impact of environmental self-identity on sustainable purchase behavior [[34], [35], [36]], and some claimed that environmental self-identity may also act as a mediator to enhance sustainable purchase behavior [28]. Despite the considerable breadth of research, there remains a notable gap in the scientific landscape regarding the moderating influence of environmental self-identity in shaping consumer intentions concerning organic food purchases. This disparity underscores the need for an ongoing investigation to improve our understanding of the complex impacts of environmental self-identity on consumer behavior in the specific setting of organic food consumption.

Recent studies have concentrated on the determinants influencing purchase intentions toward organic food [15,37]. Nonetheless, the researchers focused on examining purchase intentions associated with organic food in broader population samples, resulting in a lack of reviewing divergences between different groups. However, an emerging trend in contemporary research concerns integrating socio-demographic characteristics into analytical frameworks, representing a burgeoning attempt to examine potential differences between groups in sustainable consumption [38,39]; there remains a significant research gap in applying multi-group analysis (MGA) to more comprehensively study the SEM regarding the purchasing intentions of different consumer groups related to organic food. Therefore, more academic efforts are needed to thoroughly explore and compare how consumer segments perceive and articulate their purchase intentions for organic food.

The foremost objective of this study is to gain comprehensive theoretical insights by analyzing how consumer values and the moderating influence of environmental self-identity together shape the purchasing behavior of organic food consumers in Lithuania (Baltic economy). The research strives to answer the following key research questions.

• To what extent do consumer values, as conceptualized by the consumption values theory, influence organic food consumers' purchase intentions in Lithuania's Baltic economy?
• How does environmental self-identity moderate the relationships between consumer values and purchase intention for organic food among consumers?
• What are the variations in purchase intentions for organic food among different groups of consumers, and how can multi-group analysis (MGA) be effectively employed to further analyze the structural equation model and explore these differences?

## 2 Model conceptualization and hypothesis development

**Fig. 1:**
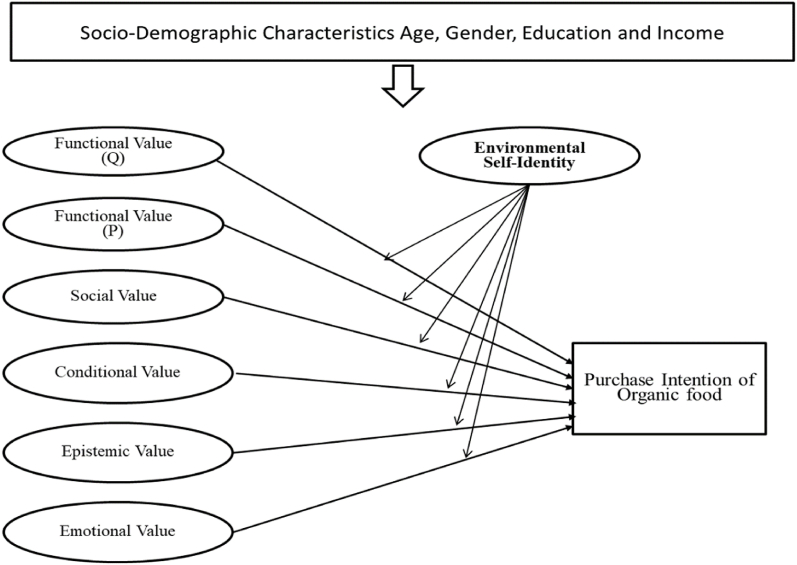
Research model.

The existing literature provides extensive evidence of values' influence on environmentally friendly decisions and behavior [40]. Alternatively, academic research emphasizes adopting a multidimensional conceptual framework of consumer values when predicting consumer choice rather than using a one-dimensional approach [41,42]. Sheth, Newman, and Gross [43] formulated and introduced a consumer value theory. According to this theoretical framework, behavioral outcomes are shaped by a quintet of values: functional, social, conditional, emotional, and epistemic. Numerous studies have shown that sustainable consumption behavior is influenced by this theory [28,44]. The conceptual framework shown in Fig. 1 was formulated to examine the complex dynamics through which consumption values influence consumers' purchase intention of organic food. Our main objective is identifying determinants underlying consumer purchasing intentions toward organic food consumption. This endeavor is accomplished by fusing the tenets of consumer value theory with the construct of environmental self-identity. Furthermore, an additional hypothesis is put forward, suggesting that the interrelationship between consumer values (functional, social, conditional, epistemic, and emotional dimensions) and purchase intention of organic food is subject to modulation by the moderating role of environmental self-identity.

### 2.1 Functional value and purchase intention

Product performance evaluation, which includes durability, endurance, dependability, dependability, price, and quality, significantly influences consumer preference for sustainable products. This evaluation of functional value is of great significance in shaping consumer decision-making processes [45]. Functional value can be categorized into quality and price components [28,45]. Consumers always pay attention to the lowest possible costs with supreme benefits and avoid frequent purchases of overpriced products [46]. Consumers prefer organic food products because of their unique features, such as natural ingredients, organic ingredients, and health benefits [47]. In addition, consumers also recognize the effectiveness of organic foods by their ability to generate economic value. Aligning the price invested in organic products with the resulting benefit increases their propensity to choose organic food [48]. Consequently, quality and price are key elements in consumer decision-making when choosing organic food. Based on this discourse, the researchers articulated the following hypotheses.

Hypothesis 1 (a) Functional Value (Q) positively influences consumer purchase intention of organic food. b. Functional value (P) positively influences consumer purchase intention of organic food.

### 2.2 Social value and purchase intention

Social influence exercised through social pressure and peer reviews plays a fundamental role in shaping consumer choice behavior [49]. In the preceding literature, social value includes factors such as self-esteem, social image, identification, manifestation of personality, and aspiration to belong to a particular social class [50]. In organic food, social value refers to the subjective assessment of the overall benefits gained from using it, considering the influence of societal norms and the pursuit of high social status. Social pressure stimulates consumers’ propensity to embrace organic foods [51]. Recently, Qasim et al. [28] and Kabir and Islam [13] found a statistically insignificant impact of social value on consumers' behavioral intentions toward organic food. Recently Desai et al. [52] found that social value has a significant on consumer choice concerning organic food. Given the uncertain results of existing research, it becomes clear that a broader distribution of studies is essential to fully understand the precise link between social value and behavioral intention. Against this background, the following hypothesis was formulated in the present study.

Hypothesis 2 Social value positively influences consumers ‘purchase intention of organic food.

### 2.3 Conditional value and purchase intention

Conditional value describes the benefits decision-makers expect from choosing a particular alternative, depending on specific situations and contextual conditions [43]. These circumstances include external factors such as timing considerations, spatial attributes, contextual nuances, promotional incentives, and cash rebates that influence consumer purchasing behavior across product categories [53]. Beyzavi and Lotfizadeh [54] showed that conditional value had nothing to do with customer choice behavior; certain prior research has directed that conditional value strongly predicts sustainable products [32,42]. In the case of organic food, discounts, and cash rebates increase affordability and create urgency, positively influencing behavioral intention toward organic food [28,55]. Based on the previous literature, the following hypothesis developed.

Hypothesis 3 Conditional value positively influences consumers' purchase intention of organic food.

### 2.4 emotional value and purchase intention

2.4

Emotional value establishes an essential of consumer values alongside functional, conditional, and social dimensions. Consumers' responses to protecting the environment and the community's well-being can influence green purchase decisions. Emotional value refers to the satisfaction resulting from the feelings or sentimental states that a product evokes [56]. Emotional values take a leading role in the integration of environmental initiatives [32]; diverse kinds of emotions, such as individual safety [56], guilt [57], and generativity, have been shown to have a significant influence on purchasing behavior and encourage consumers to make more sustainable purchases [58]. Several studies in sustainable literature found that emotional value influences consumer intention to choose sustainable products [28,32,55]. So, based on the preceding literature, the following hypothesis is established.

Hypothesis 3 Emotional value positively influences consumers’ purchase intention of organic food.

### 2.5 Epistemic value and purchase intention

Epistemic value states the “perceived utility derived from an alternative's ability to arouse curiosity, offer novelty or satisfy a desire for knowledge” [43]. According to Sharma et al. [59], consumer studies recognize that knowledge is authoritative throughout decision-making. When consumers come across a novel product, they judge its value by drawing parallels with items they are already familiar with. This cognitive process helps decide whether to proceed with the purchase. Consumers' propensity to acquire knowledge about relevance, product innovation, and attributes can substantially impact their behavioral intentions [60]. Previous research has communicated remarkable positive effects of epistemic value on the consumer purchasing behavior of sustainable products [28,54]. Hence, the following hypothesis is developed.

Hypothesis 4 Epistemic value positively influences consumers ‘purchase intention of organic food.

### 2.6 Moderating influence of environmental self-identity

Research on consumer identity is significant for shaping pro-environmental behavior [61]. Previous research has demonstrated the importance of consumer identity in assessing environmental attitudes, behaviors, and preferences [62]. Accordingly, environmental self-identity is characterized by the degree to which individuals see themselves as embodying environmentally conscientious qualities [35]. The more intense the self-identity towards the environment, the greater the individual's propensity to engage in a diverse range of environmentally friendly behaviors [63]. People with a solid environmental self-identity are more likely to see the connection between their actions and environmental degradation than those with a less pronounced environmental self-identity [64]. As an outcome, consumers’ environmental self-identity is expected to strengthen their propensity for sustainable consumption, such as purchasing environmental friendly products [65]. Earlier studies have shown that environmental identity impacts participation in pro-environmental activities [34,35] and the formation of pro-environmental beliefs [66]. Kim et al. [67] found environmental cues significantly increased the likelihood that individuals would be perceived as environmentally conscious. This suggests that when environmental factors are most important, people are motivated to choose products consistent with pro-environmental benefits.

As part of the current study, the researchers propose a constructive link between consumer values and consumers' purchase intention of organic food. In addition, a significant moderating influence of environmental self-identity can be observed, highlighting the positive association link between consumer values and purchase intention of organic food. For example, it is reasonable to assume that individuals with a stronger environmental self-identity are inclined to see organic foods’ functional value (quality). This trend is due to their increased ecological awareness and commitment to sustainable choices, leading them to prioritize natural ingredients and overall product excellence. Furthermore, it can be hypothesized that individuals with a stronger environmental self-identity will be more willing to pay a higher functional value (price) for organic food because they consider the long-term benefits to the environment and their well-being equally significant. Considering the social dimension, it is sensible to hypothesize that individuals with a stronger environmental self-identity will assign higher social value to organic food, as their purchasing decisions are likely to be influenced by their desire to be perceived as environmentally responsible by others.

Additionally, it can be posited that the relationship between conditional value (e.g., convenience, discount, subsidies), epistemic value (e.g., knowledge, information, novelty), emotional value (e.g., enjoyment, well-being), and purchase intention of organic food will be strengthened among individuals with a stronger environmental self-identity. This is because their environmental values and identity are likely to amplify their importance to these value dimensions, making them more likely to choose organic food based on convenience, knowledge acquisition, emotional satisfaction, and personal fulfillment. Based on similar considerations, modern scholarly discussions recognize that consumer self-identity plays a moderating role in the relationship between consumers' motivations/values and their actions concerning sustainable products [32]. This study provides a basis for further empirical investigations into the moderating role of environmental self-identity in understanding the complex dynamics between different dimensions of value and intention to purchase organic food.

H6 Environmental self-identity moderates the association of purchase intention of organic food with (a) function value (quality), (b) function value (price), (c) social, (d) conditional, (e) emotional, and (f) epistemic value, respectively.

#### 2.6.1 Socio-demographic characteristics

Preceding studies have recommended that demographic variables should be considered when analyzing consumer behavior. The main socio-demographic variables include age, gender, education, and income. Zhu et al. [68] explored how men and women differ in their intentions to purchase agricultural products through live streaming. They used a method called multi-group analysis to compare these differences between the sexes. Recently, Li and Zhang [69] compared consumer intention to use car sharing among different consumer groups based on gender, education, income, and age. In addition, many previous studies have considered socio-demographic variables as key determinants of individual behavior [70,71]. Therefore, gender, age, education, and income will be considered as the main elements of this study. By classifying and analyzing different groups according to the criteria of socio-demographic characteristics, the influence of different groups’ function value (quality), (b) function value (price), social value, conditional value, emotional value, epistemic value, and environmental self-identity in terms of organic food purchase intention can be compared.

## 3 Methodology

### 3.1 Data collection and survey instrument

As part of this research, a survey questionnaire was created, tested, and distributed digitally among people over 18 in Lithuania. Using a talented online research panel of approximately 20,000 willing participants, data collection is based on Computer Assisted Web Interviewing (CAWI) and a pre-screening process to certify respondent engagement. A stratified quota sampling method was used to obtain a representative sample reflecting the population of Lithuania. This approach selected approximately 1000 potential respondents who received online questionnaires and were divided into two subgroups (strata) based on gender and age (18–65), with proportions consistent with those of the general population. A random sample was drawn from each stratum. The survey was distributed to 2887 respondents and yielded 1000 useable responses, supporting the goal of a representative sample through stratified randomization. This method differs from traditional probability sampling and is preferred because it produces more comparable data [72].

### 3.2 Measures

An empirical study used a designed questionnaire to collect data, starting with an English version later translated into Lithuanian by two independent specialist translators to ensure conceptual equivalence. The questionnaire contained two parts: In the main part, the demographic data of the respondents, such as age group, income, gender, and education, were recorded. The next part focused on measuring different factors such as functional value (quality and price), social value, emotional value, conditional value, epistemic value, environmental self-identity, and organic food purchase intention (See Table 1).

**Table 1:** Respondent details.

Demographics		Frequency
Gender	Female	508
	Male	487
	Other	5
	Don't want to answer	0
	18–24	117
Age	25–34	324
	35–44	333
	45–54	226
	Basic Primary Education	13
	Secondary Education	120
Education	Higher Secondary Education and Special Education	177
	College Education	28
	Higher education (non-university level)	160
	Higher Education (University level)	502
	Less than 350 EUR	32
	351 - 450 EUR	40
	451 - 550 EUR	50
	551 - 750 EUR	235
	751 - 950 EUR	129
Income	951 - 1500 EUR	187
	1501 - 2000 EUR	76
	2001 - 2500 EUR	21
	2501 - 3000 EUR	11
	3001 - 4000 EUR	5
	4001 More Than	12
	Don't want to answer	202

Items for measuring functional value (Quality) taken from the studies of Biswas and Roy [31] and Qasim et al. [28], Items to measure functional value (Price), conditional value, social value, and emotional Items adapted from Lin and Huang [73], epistemic value items adopted from the study of Suki et al. [30], environmental self-identity items adopted from Van et al., [35]. Last but not least, Items to measure the purchase intention of organic food were adopted from the study by Kabir and Aslam [13]. All items were evaluated using a 5-point Likert scale, where respondents could express their degree of agreement on a range from “strongly disagree” (1) to “strongly agree” (5). Please see Appendix 1.

## 4 Data analysis

In this study, SPSS and SmartPLS were used to examine the data. SPSS was used to identify multivariate outliers and measure common method bias. PLS-SEM was the most appropriate analysis tool to validate the existing theoretical framework. The analysis was performed in two steps. The degree of model fit was initially examined, including reliability and validity. After that, the proposed hypotheses were tested using the 2000 bootstrapping method.

### 4.1 Common method variance

Data cleansing methods were implemented to mitigate the potential impact of common method biases on dataset credibility. The presence of common method biases poses a significant challenge to the reliability of the data [74]. It was, therefore, essential to ensure the independence of the data from such biases. To achieve this, Harman's one-factor test was used as a diagnostic tool to detect common method bias. According to this assessment, if a singular factor accounts for less than 50 % of the total variance in the data set, there is no general methodological error. The outcome of the analysis discovered that a single factor accounted for only 32.05 % of the variance, endorsing the absence of any significant common method bias.

### 4.2 Measurement model

Convergent validity was appraised through factor loadings, average variance (AVE) and composite reliability (CR), as outlined in Table 2. Factor loadings showed strong convergent validity, with the value ranging from 0.739 to 0.928, according to the criteria mentioned by Fidell [75]. All the CR values ranged from 0.817 to 0.938, and AVE values from 0.620 to 0.834, meeting the consensus and reliability stated by Hair et al. [76]. These outcomes prove the reliability and validity of the measures used in the present study.

**Table 2:** Factors loading, composite reliability, and average variance extracted.

Constructs	Items	Loadings	CR	AVE
Functional Value (Quality)			0.854	0.662
	FV(Q)1	0.792		
	FV(Q)2	0.804		
	FV(Q)3	0.844		
Functional Value (Price)			0.866	0.620
	FV(p)1	0.818		
	FV(p)2	0.900		
	FV(p)3	0.861		
Social Value			0.929	0.813
	SV1	0.896		
	SV2	0.910		
	SV3	0.899		
Conditional Value			0.817	0.698
	CV1	0.831		
	CV2	0.739		
	CV3	0.748		
Emotional Value			0.938	0.834
	EV1	0.924		
	EV2	0.928		
	EV3	0.886		
Epistemic Value			0.866	0.620
	EP1	0.813		
	EP2	0.870		
	EP3	0.818		
Environmental Self-Identity			0.920	0.794
	SE1	0.854		
	SE2	0.916		
	SE3	0.903		
Purchase Intention (Organic Food)			0.904	0.702
	PI1	0.749		
	PI2	0.865		
	PI3	0.888		
	PI4	0.843		

Note: FV (Q) = functional value (Quality); FV (P) = functional value (price); SV = social value; CV = conditional value; EV = emotional value; EP = epistemic value; ESI = environmental self-identity; PI = purchase intention of organic food.

### 4.3 Discriminant validity

The criteria recommended by Fornell and Larcker [74] were used to gauge the discriminant validity. Specifically, it was ensured that the square root of the AVE for each indicator exceeded the total correlation of the construct with any other constructs. As revealed in Table 3, the results confirmed that all constructs met this requirement. In addition, the proportion of heterotrait correlations (HTMT) was analyzed using the HTMT criteria 0.90 proposed by Henseler et al. [77]. The results in Table 3 indicated that all HTMT values for building relationships were below the threshold of 0.90, thus satisfying the criteria for HTMT.90. Consequently, it can be concluded that the discriminant validity conditions were adequately met for all study constructs.

**Table 3:** Co-relation and HTMT ratio.

Fornell-Larcker Criterion								
	CV	EP	ES	EV	FV(P)	FV(Q)	PI	SV
CV	0.773							
EP	0.368	0.787						
ES	0.343	0.335	0.891					
EV	0.599	0.414	0.379	0.913				
FV(P)	0.489	0.350	0.242	0.532	0.860			
FV(Q)	0.505	0.267	0.337	0.512	0.608	0.813		
PI	0.566	0.424	0.523	0.516	0.440	0.509	0.838	
SV	0.451	0.320	0.150	0.548	0.500	0.373	0.292	0.902
HTMT Ratio								
CV								
EP	0.494							
ES	0.448	0.403						
EV	0.784	0.483	0.425					
FV(P)	0.656	0.422	0.281	0.612				
FV(Q)	0.709	0.328	0.403	0.617	0.770			
PI	0.739	0.503	0.606	0.582	0.512	0.610		
SV	0.599	0.369	0.169	0.614	0.576	0.458	0.322	

Note: FV (Q) = functional value (Quality); FV (P) = functional value (price); SV = social value; CV = conditional value; EV = emotional value; EP = epistemic value; ESI = environmental self-identity; PI = purchase intention of organic food.

### 4.4 Structural model

The model's fitness depends on its ability to accurately predict the endogenous model's outcomes [78]. The primary measures for assessing the internal model include checking the coefficient of determination (R2) and cross-validation redundancy (Q2). These indicators are critical in determining the model's ability to predict outcomes and explain the variability within the dependent variable [78]. The R-squared (R2) gauge estimates the precision of the model's predictions by quantifying the amount of variability in the dependent variable accounted for by the independent variables [79]. The researchers classified the R-squared value into high, medium, and low classifications. R2 is significant when it exceeds 0.6, indicates a moderate level between 0.3 and 0.6, and is considered weak when below 0.3 [80]. The results show an R2 value of 51 %, indicating a high level of closeness. Another metric used to assess model quality is cross-validation redundancy (Q2). Using the blindfold technique, we evaluated the predictive significance of the internal model, with a Q2 value greater than 0 indicating the model's effectiveness in producing meaningful predictive results [79]. The Q2 value in this study is 49.8 and indicates an exceptionally high-quality level of the inner model.

To evaluate the hypothetical model, the researchers used Partial Least Squares Structural Equation Modeling (PLS-SEM) with the bootstrap 2000 sampling technique recommended by Haenlein and Kaplan (2004). Table 4 shows the hypothetical relationship results. The results indicated the FV (Q) (β = 0.173, p < 0.01), SV (β = 0.075, p < 0.05), CV (β = 0.263, p < 0.05), EV (β = 0.074, p < 0.05) and EP (β = 0.095, p < 0.05) have positive on PI. As a outcome H1(a), H3, H4, H5 and H6 are accepted. The results suggested that FV (P) (β = 0.043, p > 0.05) has a non-significant influence on the purchase intention of organic food. Hence, H1(b) is rejected.

**Table 4:** Hypothetical relationships.

Hypothesis	Relationship	Beta	T-value	P-value	Status
H1(a)	FV(Q) - > PI	0.173	5.048	0.000	Accepted
H1(b)	FV(P)- > PI	0.043	1.341	0.090	Rejected
H2	SV - > PI	0.075	2.555	0.005	Accepted
H3	CV - > PI	0.263	6.955	0.000	Accepted
H4	EV - > PI	0.074	2.046	0.020	Accepted
H5	EP - > PI	0.095	2.908	0.002	Accepted
H6(a)	ESI x FV(Q) - > PI	0.035	1.117	0.120	Rejected
H6(b)	ESI x FV (P) - > PI	0.014	0.392	0.262	Rejected
H6(c)	ESI x SV- > PI	0.013	0.419	0.254	Rejected
H6(d)	ESI x CV- > PI	0.824	7.393	0.000	Accepted
H6(e)	ESI x EV- > PI	0.564	2.101	0.018	Accepted
H6(f)	ESI x EP - > PI	0.019	0.569	0.413	Rejected

Note: FV (Q) = functional value (Quality); FV (P) = functional value (price); SV = social value; CV = conditional value; EV = emotional value; EP = epistemic value; ESI = environmental self-identity; PI = purchase intention of organic food.

**Fig. 2:**
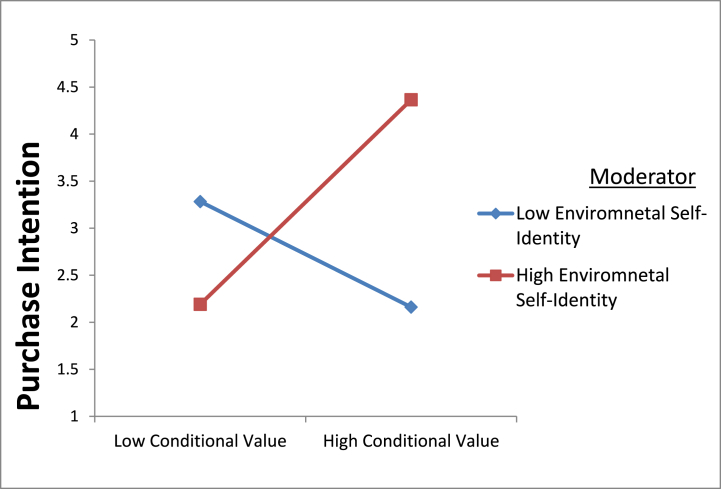
(Environmental self-identity strengthen relationship between conditional value and purchase intention of organic food).

**Fig. 3:**
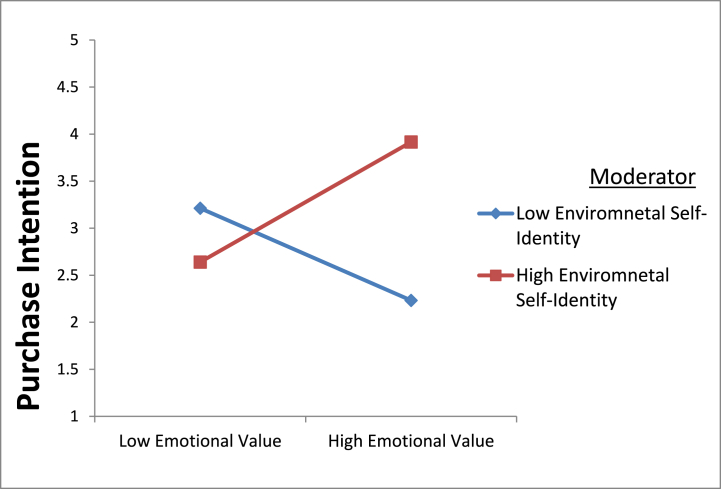
(Environmental self-identity strengthen relationship between emotional value and purchase intention of organic food).

The research also examined the role of environmental self-identity as a moderator in the relationships between different values and purchase intention. The results directed that ESI strengthens the relationship between CV and PI (β = 0.408, p < 0.01) and improves the link between EV and PI (β = 0.564, p < 0.05) for organic food. Thus, hypotheses H6 (d) and H6(e) were supported. The findings suggest that a higher environmental self-identity (ESI) strengthens the positive correlation between both conditional value and purchase intention of organic food, as well as emotional value and purchase intention of organic food. To further clarify the moderating effect, we conducted a slope analysis. Fig. 2, Fig. 3 depict, respectively, the association between CV and PI, and between EV and PI, at elevated levels of ESI. However, ESI did not moderate the relationships between SV and PI, FV (P) and PI, FV (Q) and PI, and EP and PI (β = 0.075, p > 0.05; β = 0.014, p > 0.05; β = 0.013, p > 0.05; β = 0.019, p > 0.05, respectively). Therefore, hypotheses H6 (a), H6(b), H6(c) and H6(f) were not supported.

### 4.5 Multi-group analysis

By a multi-group analysis (MGA), the study studied the influences of variables across diverse groups. The parameters examined included age, gender, income level, and level of education. According to Ref. [81], the analysis of these different groups is approached through four methodologies: primary is the parametric approach, renowned for its leniency; followed by the permutation and confidence-based approaches, distinguished for their more rigorous nature; and culminating in the most thorough technique, Henseler's multi-group approach. Notably, Henseler et al. [77] have advanced this paradigm further by introducing PLS-MGA (multi-group analysis). This comprehensive extension discerns noteworthy disparities between groups when they fall below the threshold of 0.05 or surpass the level of 0.95. This research used a percentile bootstrapping technique to scrutinize the groups' differences. The results of our analysis clearly show a remarkable variance between groups, within a 5 % margin of error, which becomes particularly evident when the p-value exceeds the 95 % threshold or falls below the 5 % mark. Significantly, the percentile below 5 % shows that Group A's bootstrap results outperform Group B, while the above 95 % shows that Group B performed better than Group A.

The pathways for each group that were analyzed are listed in Table 5. The outcomes of MGA, as indicated by the p-values, show notable differences between the groups. Regarding gender, H1 (p = 0.018 < 0.05) showed significant inequality, underscoring that the association between functional value (quality) and purchase intention was stronger in the female cohort than in the male cohort Cohort. Likewise, H6(d) (p = 0.039 < 0.05) exhibited significant divergence, suggesting that environmental self-identity significantly strengthens the association between conditional value and intention to purchase organic food in the female group and surpasses their effect in the male group. Finally, our study found that H6(d) (p = 0.034 < 0.05) differed significantly, suggesting that environmental self-identity significantly strengthens emotional value and purchase intention of organic food in the female group than in the male group. There were significant differences for age H2 (p = 0.04 > 0.95), suggesting that the association between social value and purchase intention of organic food was stronger in the young group than in the older group. As we shifted our focus to the field of education, our research uncovered a striking finding in Hypothesis 6(b) (p = 0.011 > 0.05). This difference suggests that the association between functional value (quality) and purchase intention of organic food was significantly stronger among the highly educated than the less educated. Regarding income, Hypothesis 1 (a) (p = 0.001 < 0.05) showed significant variation. This suggests that the association between functional value (Q) and purchase intention of organic food showed a stronger correlation within the high-income group than in the low-income group.

**Table 5:** MGA results.

	H1(a)	H1 (b)	H2	H3	H4	H5	H6 (a)	H6 (b)	H6 (c)	H6 (d)	H6 (e)	H6 (f)
**Gender**												
Female (508)	0.111	0.089	0.069	0.265	0.097	0.089	−0.096	−0.029	0.002	−0.125	0.150	0.050
Male (487)	0.247	0.025	0.085	0.283	0.014	0.025	0.004	−0.008	0.058	0.014	0.005	−0.045
Diff	−0.136	0.064	−0.016	−0.018	0.083	0.064	−0.100	−0.021	−0.056	−0.139	0.145	0.095
PLS MGA Value	**0.018**	0.164	0.393	0.396	0.131	0.487	0.052	0.387	0.185	**0.039**	**0.034**	0.094
**Age**												
Old (473)	0.156	−0.089	0.032	0.569	−0.077	0.108	−0.007	−0.037	0.028	−0.154	0.172	0.079
Young (527)	0.166	0.017	0.228	0.580	−0.135	−0.001	−0.103	0.049	−0.106	−0.093	0.179	0.018
Diff	−0.010	−0.106	−0.196	−0.011	0.058	0.109	0.096	−0.086	0.134	−0.061	−0.007	0.061
PLS MGA Value	0.167	0.135	**0.991**	0.117	0.083	0.240	0.199	0.454	0.116	0.127	0.246	0.406
**Education**												
High Education (662)	0.200	0.050	0.040	0.255	0.076	0.115	0.026	−0.042	0.017	−0.062	0.104	0.000
Low Education (338)	0.109	0.056	0.138	0.299	0.050	0.075	−0.139	−0.049	0.094	−0.026	0.035	0.023
Diff	0.091	−0.006	−0.098	−0.044	0.026	0.040	0.165	0.007	−0.077	−0.036	0.069	−0.023
PLS MGA Value	0.104	0.466	0.055	0.293	0.371	0.265	**0.011**	0.467	0.157	0.330	0.220	0.384
**Household Income**												
High Income (312)	0.358	0.059	0.011	0.199	0.018	0.171	0.032	−0.079	−0.063	0.020	0.009	−0.079
Low Income (486)	0.061	0.046	0.099	0.294	0.095	0.102	−0.053	−0.047	0.059	−0.037	0.105	−0.047
Diff	0.297	0.013	−0.088	−0.095	−0.077	0.069	0.085	−0.032	−0.122	0.057	−0.096	−0.032
PLS MGA Value	**0.001**	0.431	0.086	0.113	0.167	0.171	0.085	0.339	0.032	0.258	0.140	0.128

## 5 Discussion

Our study found a stimulus of consumption values on the purchase intention of organic food; specifically, our findings designate a positive impact of functional value (quality) on the purchase intention of organic food, aligning with preceding research outcomes [28,82]. In contrast, functional value price has a non-significant influence on the purchase intention of organic; the results support past studies' price as a less critical factor for sustainable products [83]. This suggests that Lithuanian consumers tend to value the quality of organic food and place great value on its health-promoting and nutritious properties rather than solely focusing on the economic aspects of cheaper choices. This preference shift is due to a growing concern about the harmful effects of chemical fertilizers and pesticides used in agriculture on human well-being. Consequently, these consumers are willing to devote resources to more expensive organic products as they see it as a preventive measure to stave off potential health costs associated with consuming less expensive non-organic alternatives. Social value has a significant influence on the purchase intention of organic food. The findings are dependable, with preceding findings endorsing that social pressures encourage consumers to consume organic foods [55,82]. Research results show that the opinions and recommendations of family members, relatives and friends primarily determine consumers' decisions about purchasing organic food in Lithuania. This highlights the significant impact of social influence on purchase intentions for organic food in Lithuania. Although Lithuania is generally recognized as an individualistic society, this shows the positive influence of social factors on consumer preferences for organic food. This attitude is reflected in the observations of Liobikienė et al. [10], who emphasize the important role of family, relatives and friends in influencing the purchase of sustainable products in Lithuania. Our study identified a notable impact of conditional value on purchase intention of organic food, a finding that confirms previous research [28,48]. The finding suggests that conditional values allure Lithuanian consumers to purchase organic food. As an outcome, conditional value in terms of seller's promotional offers and government subsidies may influence consumers' purchase intention of organic food products. Emotional value has a significant influence on the purchase intention of organic food. The study's findings reveal that organic food consumption in Lithuania elicits favorable emotional responses among consumers, subsequently motivating them to adopt ecologically conscientious behaviors.

Moreover, nurturing an emotional bond with the natural surroundings encourages the enhancement of an individual's environmental consciousness, thereby exerting a noticeable impact on their choices regarding purchasing organic food products. This proclamation is consistent with preceding research [28,73], emphasizing the connection between emotional engagement with the environment and pro-environmental behaviors. However, it deviates from current research that directed a lack of significant influence of emotional value on consumer preferences for sustainable products [32]. Epistemic value positively influences the purchase intention of organic food; the findings support previous studies [28,32]. The epistemic value primarily affects the knowledge and innovation aspects embodied in the products. The study emphasizes that consumers are more likely to accept organic food consumption if they understand the environmental issues associated with non-organic alternatives. This trend can be attributed to the appeal of unique aspects of organic foods, including cultivation methods and reduced reliance on synthetic additives. As consumers gain insight into these unique characteristics, a sense of novelty emerges, further strengthening their relationship with organic food consumption.

In the Lithuanian context, this study examined the role of consumers' ecological self-identity as a moderator in the links between consumer values and purchase intention of organic food. The study's results revealed a significant positive moderating effect of environmental self-identity on the relationship between emotional and purchase intention with organic food. This finding suggests that an individual's commitment to environmental well-being strengthens the link between emotional value and propensity to purchase organic food. This phenomenon can be outlined as how consumers' ecological principles act on their emotional feelings and spur them to environmentally friendly actions, including adopting organic foods. Furthermore, the results of the moderation analysis showed that environmental self-identity also plays a central and statistically significant moderating role in shaping the relationship between conditional value and purchase intention of organic food. This observation can be explained by recognizing that consumers' environmental values prompt them to respond in an environmentally friendly manner when exposed to various stimuli associated with consuming organic food. Consequently, consumers' environmental self-identity and environmental considerations can strengthen the link between conditional value and purchase intention of organic food. However, it is essential to note that no significant moderating effect of environmental self-identity was found regarding the associations between social value, functional value (including quality and price), and epistemic value with the purchase intention of organic food. These aspects require further investigation by future researchers to gain deeper insights into the complex interplay underlying organic food consumer purchase behavior in the Lithuanian context.

This research shows clear differences in purchase intentions due to differences in demographics such as gender, age, educational background, and income level. Concerning gender, the association between functional value (quality) and purchase intention of organic food is statistically significant, particularly in female cohorts. The research revealed that, much like their global counterparts, female consumers in Lithuania tend to attribute significant importance to functional value aspects such as health benefits and nutritional content when deciding to buy organic food. This inclination toward functional benefits aligns with the observed significance of the relationship between functional value quality and purchase intentions among female consumers. Our findings corroborate previous research indicating that women prefer consuming organic food due to their belief in its greater health benefits for themselves and their families [84]. Our research has revealed that among Lithuanian women, there is a notable strengthening of the connection between environmental self-identity and conditional value factors (such as convenience, discounts, and subsidies) in influencing the intention to purchase organic food. With statistical significance, this enhancement underscores the pivotal role of environmental self-identity in shaping organic food purchasing decisions. This aligns with previous research emphasizing the influential role of environmental self-identity in guiding sustainable consumption behaviors [34]. Lithuanian women, akin to counterparts in other societies, demonstrate heightened environmental awareness and ethical connections, which magnify the impact of conditional value factors on their purchase intentions [85]. Our study found that environmental self-identity strengthens the relationship between emotional value and purchase intention for organic food among women. Their environmental self-identity influences this effect. This is in line with the cultural norm of Lithuania, where women hold key roles in household stewardship and have strong emotional ties to family well-being. Their traditional caring responsibilities lead them to prioritize the well-being of their loved ones, making them more responsive to the emotional aspect of environmentally conscious choices.

For age, our research similarly revealed that environmental self-identity plays a vital role in amplifying the association between social value and purchase intention of organic food among young individuals, and this relationship demonstrated statistical significance. This phenomenon can be attributed to the increased exposure of young people to social media platforms, which strongly influences their perceptions of social value and consumption behaviors [86]. The ubiquitous presence of social media in the lives of young Lithuanians exposes them to a wide range of socially conscious content, campaigns, and influencers. It shapes their perception of the social benefits associated with sustainable consumption. This strengthens the alignment of their self-identity with environmental values, increasing the influence of social values on their intention to engage in green purchasing decisions. In terms of education, our investigation has unveiled a notable revelation: a strong environmental self-identity distinctly enhances the connection between the functional value (quality) of organic food products, ultimately influencing the intention to purchase organic food. This link has been empirically verified as significant among individuals with higher levels of education. This discovery aligns with prior research that underscores the role of personal environmental identity in shaping consumers' preferences for eco-friendly items [33]. The reason behind this finding can be attributed to the high awareness and rigor of educated individuals in Lithuania. Their environmental self-identity reinforces their concern for functional attributes and qualities when considering organic food options. The alignment between their values and purchasing decisions was most pronounced in the higher education cohort, where integrating identity with product attributes drove the intention to purchase organic food. In terms of income, a remarkable result emerges from the statistical analysis, highlighting the importance of the connection between functional value (quality) and purchase intention of organic food, especially in the affluent household segment. This finding sheds an eye-opening spotlight on how affluent Lithuanian consumers approach organic food choices. This observation is consistent with previous research showing a positive correlation between higher income levels and an increased propensity for organic products [87]. This propensity is likely rooted in individuals with more significant disposable income's tendency to prioritize health and environmental concerns, leading them to prefer organic alternatives due to perceived healthiness and ecological friendliness [88]. The growing awareness of health and environmental issues is fuelling the increase in the adoption of organic food in Lithuania [89]. The inclination of the high-income group towards organic foods can be attributed to their ability to afford premium prices, often associated with better quality and sustainable production methods.

## 6 Conclusions

### 6.1 Theoretical contribution

This research makes a significant and notable contribution to the established knowledge of intentional behavior, effectively increasing and improving its scientific basis. This study introduces a unique and previously unexplored research context by explicitly focusing its investigation on organic food in the Baltic economy, specifically in Lithuania. This distinctive research setting, distinct from previous inquiries, creates a new analytical perspective that enriches the theoretical framework. In addition, the empirical validation of TCV [43] in the context of organic food behavior in Lithuania accentuates the scope and applicability of this theoretical framework.

Secondly, this study explores the moderating capacity of environmental self-identity in the link between consumption values and purchase intention of organic food. Previous research has integrated environmental self-identity as a precursor to influencing attitudes and purchase intentions toward environmental behaviors [34,64]. The results of this study further confirm the importance of environmental self-identity and advocate its identification as a moderator within the consumption values framework, enhancing the comprehensive investigation of purchase intentions and consumer behavior.

Thirdly, this study's findings show a significant association in purchasing organic food, together with the results from the study's prior research using multi-group analysis [45,84]. The combined application of Partial Least Squares Statistical Modeling (PLS-SEM) and the Multi-Group Analysis (MGA) technique proved very effective. This combination not only identifies individual behaviors but also accounts for the different behaviors of different groups. In simple terms, this approach allows us to study behavior and measure how other groups behave differently.

### 6.2 Managerial and practical implications

The results of our study offer valuable insights for marketers operating in the organic food market in Lithuania. It turns out that several factors have a significant influence on consumers' purchasing intentions for organic food. These factors include functional value (quality), social value, conditional value (convenience, discounts, subsidies), epistemic value (knowledge, information, novelty), and emotional value. To capitalize on these insights, marketers should adjust their strategies to align with the diverse values driving organic food consumption. Different consumer groups can be effectively addressed by highlighting organic products' high quality and unique characteristics, their social and environmental benefits, and offering targeted promotions or convenient purchasing options. By aligning marketing efforts with identified consumer values, companies can encourage increased purchase intention and build a loyal customer base.

Our research has significant implications for policymakers promoting sustainable and health-conscious dietary choices in Lithuania. The observed impact of environmental self-identity as a facilitator underscores the importance of nurturing individuals' connection to their environmentally conscious selves. Policymakers can support this link through campaigns and initiatives that promote environmental responsibility and ultimately increase the impact of conditional value factors such as convenience, rebates, and subsidies on organic food purchase intentions. Developing policies encouraging companies to offer more accessible and affordable organic options can incentivize consumers to make green choices. By linking policy initiatives to consumption values identified in our study, Lithuania can advance its goals of sustainable consumption and a healthier population.

In practical terms, these findings empower businesses to refine their approach and cater to the diverse preferences of different consumer segments. Using a segmentation strategy based on identified consumption values – functional, social, conditional, epistemic, and emotional – can enable companies to create targeted marketing campaigns and design products that resonate with different groups. Collaborating with environmental organizations to raise awareness of sustainable practices and strengthen the link between consumer self-identity and green choices can be very effective. In addition, it offers educational resources that emphasize the benefits of organic food and the influence of epistemic values, cultivating a well-informed consumer base. These practical applications can yield positive results for businesses and society's more expansive pursuit of sustainable consumption.

### 6.3 Limitations and future research directions

Our research addressed the complexity of organic food purchase intentions in Lithuania. We found that various consumer values play a significant role, and the influence of environmental self-identity is notable. The practical implications of our study extend beyond its immediate scope and provide valuable insights that marketers, policymakers, and businesses can apply. However, it is essential to acknowledge the limitations of our study. Further research is required to gain a deeper understanding of these relationships. Future research should consider additional variables such as cultural characteristics and economic factors to capture patterns in organic food consumption comprehensively. By continually researching these aspects, managers can improve their strategies, policies, and interventions, contributing to a more sustainable future in Lithuania and beyond. Our study could serve as a basis for cross-cultural research in Baltic countries such as Latvia and Estonia. Such research could provide an in-depth understanding of the diverse cultural, social, and economic dynamics in these markets and provide valuable insights to professionals and marketers in the global and regional business landscape.

## Data availability statement

Data will be made available on request.

## CRediT authorship contribution statement

**Aušra Rūtelionė:** Writing – review & editing, Validation, Supervision, Software, Resources, Project administration, Formal analysis, Data curation, Conceptualization. **Muhammad Yaseen Bhutto:** Writing – original draft, Visualization, Validation, Software, Methodology, Investigation, Formal analysis, Conceptualization.

## Declaration of competing interest

The authors declare the following financial interests/personal relationships which may be considered as potential competing interests: Muhammad Yaseen Bhutto reports financial support, administrative support, article publishing charges, equipment, drugs, or supplies, statistical analysis, and travel were provided by Research Council of Lithuania (LMTLT). Muhammad Yaseen Bhutto reports a relationship with Research Council of Lithuania (LMTLT) that includes: funding grants. Muhammad Yaseen Bhutto has patent issued to S-PD-22–67. No conflict of interest If there are other authors, they declare that they have no known competing financial interests or personal relationships that could have appeared to influence the work reported in this paper.
