# Supplementary material for: "Eco-conscious appetites: Investigating organic food purchase intentions through consumption values, empowered by environmental self-identity and analyzed using MGA – Baltic insights"
Source: Heliyon. 2024 Jul 27;10(15):e35330. doi: 10.1016/j.heliyon.2024.e35330 (PMC11336583; doi:10.1016/j.heliyon.2024.e35330)
Supplement: Multimedia component 1 [file mmc1.docx]

**Questionnaire on Sustainability in the Food Industry and Organic Food Consumption in Lithuania**

Thank you for participating in this research study. Your feedback is invaluable in understanding the significance of sustainability in the food industry and enhancing our endeavors to foster positive environmental and social outcomes.

| 1. **Gender** |
| --- |
| - Female |
| - Male |
| - Other |
| - Don't want to answer |
| 1. **Age** |
| - 18-24 |
| - 25-34 |
| - 35-44 |
| - 45-54 |
| 1. **Education** |
| - Basic Primary Education |
| - Secodary Education |
| - Higher Secondar Education and Sepecial education |
| - College Education |
| - Higher Education (non-university level) |
| - Higher Education (University level) |
| 1. **Income** |
| - Less than 350 EUR |
| - 351 - 450 EUR |
| - 451 - 550 EUR |
| - 551 - 750 EUR |
| - 751 - 950 EUR |
| - 951 - 1500 EUR |
| - 1501 - 2000 EUR |
| - 2001 - 2500 EUR |
| - 2501 - 3000 EUR |
| - 3001 - 4000 EUR |
| - 4001 More Than |
| - Don’t want to answer |

Scale: (1=Strongly Disagree) (2= Disagree) (3=Neither agree not disagree) (4=Agree) (5=Strongly agree)

| **Construct** | **Items** | **1** | **2** | **3** | **4** | **5** |
| --- | --- | --- | --- | --- | --- | --- |
| **Functional Quality** | The organic food product has expectable standard quality |  |  |  |  |  |
|  | The organic food product is made from non-hazardous substances. |  |  |  |  |  |
|  | Taste of organic food is good. |  |  |  |  |  |
| **Functional Price** | The organic food product is reasonably priced. |  |  |  |  |  |
|  | The organic food product offer value for money. |  |  |  |  |  |
|  | The organic food product has a good economic value. |  |  |  |  |  |
| **Social Value** | Purchase of organic food product will help me gain social approval. |  |  |  |  |  |
|  | Purchase of organic food product will make a positive impression on other people. |  |  |  |  |  |
|  | Purchase of organic food product will improve the way I am perceived |  |  |  |  |  |
| **Conditional Value** | I would buy organic food products if they were easy to acquire (accessible nearby). |  |  |  |  |  |
|  | I would buy the organic food product instead of conventional products under worsening environmental conditions. |  |  |  |  |  |
|  | I will purchase organic food products over conventional substitutes if they are offered at a subsidized rate |  |  |  |  |  |
| **Emotional Value** | Buying the organic food product instead of conventional products would feel like making a good personal contribution to something better |  |  |  |  |  |
|  | Buying the organic food product instead of conventional products would feel like the morally right thing |  |  |  |  |  |
|  | Buying the organic food product instead of conventional products would make me feel like a better person |  |  |  |  |  |
| **Epistemic Value** | Before buying the organic food, I would obtain substantial information about the different makes of products |  |  |  |  |  |
|  | I would acquire a great deal of information about the different makes before buying the organic food.” . |  |  |  |  |  |
|  | I am willing to seek out novel information before buying the organic food . |  |  |  |  |  |
| **Environmental Self-identity** | Acting environmental friendly is an important part of who I am. |  |  |  |  |  |
|  | I am the type of person who acts environmental friendly. |  |  |  |  |  |
|  | I see myself as an environmental-friendly person. |  |  |  |  |  |
| **Purchase Intention of Organic Food** | If organic foods are affordable, I am willing to buy them |  |  |  |  |  |
|  | I am prepared to purchase organic foods |  |  |  |  |  |
|  | If organic foods are available to buy, I want to buy them. |  |  |  |  |  |
|  | I prefer to eat organic foods |  |  |  |  |  |
